# The Human Pancreas Proteome Defined by Transcriptomics and Antibody-Based Profiling

**DOI:** 10.1371/journal.pone.0115421

**Published:** 2014-12-29

**Authors:** Angelika Danielsson, Fredrik Pontén, Linn Fagerberg, Björn M. Hallström, Jochen M. Schwenk, Mathias Uhlén, Olle Korsgren, Cecilia Lindskog

**Affiliations:** 1 Science for Life Laboratory, Department of Immunology, Genetics and Pathology, Uppsala University, Uppsala, Sweden; 2 Science for Life Laboratory, KTH - Royal Institute of Technology, Stockholm, Sweden; Centro Nacional de Investigaciones Oncológicas (CNIO), Spain

## Abstract

The pancreas is composed of both exocrine glands and intermingled endocrine cells to execute its diverse functions, including enzyme production for digestion of nutrients and hormone secretion for regulation of blood glucose levels. To define the molecular constituents with elevated expression in the human pancreas, we employed a genome-wide RNA sequencing analysis of the human transcriptome to identify genes with elevated expression in the human pancreas. This quantitative transcriptomics data was combined with immunohistochemistry-based protein profiling to allow mapping of the corresponding proteins to different compartments and specific cell types within the pancreas down to the single cell level. Analysis of whole pancreas identified 146 genes with elevated expression levels, of which 47 revealed a particular higher expression as compared to the other analyzed tissue types, thus termed pancreas enriched. Extended analysis of *in vitro* isolated endocrine islets identified an additional set of 42 genes with elevated expression in these specialized cells. Although only 0.7% of all genes showed an elevated expression level in the pancreas, this fraction of transcripts, in most cases encoding secreted proteins, constituted 68% of the total mRNA in pancreas. This demonstrates the extreme specialization of the pancreas for production of secreted proteins. Among the elevated expression profiles, several previously not described proteins were identified, both in endocrine cells (CFC1, FAM159B, RBPJL and RGS9) and exocrine glandular cells (AQP12A, DPEP1, GATM and ERP27). In summary, we provide a global analysis of the pancreas transcriptome and proteome with a comprehensive list of genes and proteins with elevated expression in pancreas. This list represents an important starting point for further studies of the molecular repertoire of pancreatic cells and their relation to disease states or treatment effects.

## Introduction

The pancreas is a composite organ with two main and completely diverse functions: enzyme production for digestion and hormone secretion for regulation of blood glucose levels. The specialized functions are dependent on the molecular repertoire of the cell types building each compartment. Most of the pancreas is composed of exocrine tissue consisting of acinar and ductal cells, whereas the scattered islets of Langerhans with endocrine function constitute only 1–2% of the total organ mass. In addition to the specific pancreatic cell types, the heterogeneous pancreatic tissue is also highly vascularized and innervated, especially within the islet compartment.

The development of gene expression profiling techniques has facilitated the characterization of tissues to determine the link between expressed genes and the phenotype and function of the various cell types within an organ. By exploring the transcriptome, insights into cell type specificity and normal functions as well as pathological conditions can be provided. Transcriptomics analyses provide the means to compare different tissue types and to identify genes with expression restricted to certain cell and tissue types. The insulin-producing beta cells in pancreatic islets are of particular interest since they are affected in type 1 and type 2 diabetes (T1D/T2D), but detailed characterization of the transcriptome in the exocrine compartments is also of high relevance, in order to further understand digestion and the underlying molecular mechanisms of pancreatic disease, e.g. pancreatitis and pancreatic cancer.

Despite the obvious advantages of gene expression analysis, interpretation of acquired transcriptomics data is a challenge due to the heterogeneous nature of complex tissues. The presence of gene-products on the transcript level in a specific cell type or tissue can be assessed by quantifying the transcripts using next generation sequencing technology (RNA-Seq) [1]. Here we have analyzed genes expressed in normal human pancreas and these data were compared with the transcriptome of 26 other human tissue types based on recently published RNA-seq data [2]. The analyses here also include isolated islet and exocrine preparations derived from organ donor patients. The transcriptomics approach was combined with antibody-based protein profiling using tissue microarrays (TMAs) and immunohistochemistry, in order to create a comprehensive knowledge resource of identified proteins localized in defined compartments of the pancreas, such as islets of Langerhans, exocrine glandular cells and ductal cells.

## Material and Methods

### Tissue samples

Human tissue samples used for protein and mRNA expression analyses were collected and handled in accordance with Swedish laws and regulation and obtained from the Department of Pathology, Uppsala University Hospital, Uppsala, Sweden, as part of the sample collection governed by the Uppsala Biobank (http://www.uppsalabiobank.uu.se/en/). All human tissue samples used in the present study were anonymized in accordance with approval and advisory report from the Uppsala Ethical Review Board (Dnr Ups 02-577 (protein) and Dnr 2011/473 (RNA)), and consequently the need for informed consent was waived by the ethics committee. Fresh frozen tissue from 27 different histologically normal tissue types were included as previously described [2] including two pancreatic samples obtained from one female (individual 1, 59 years old) operated for a microcystic adenoma and one male (individual 2, 60 years old) who received surgery for a neuroendocrine tumor. Cryosections from both tissue samples showed normal histology without any contamination of tumor cells. Isolated pancreatic islets and exocrine tissue were obtained from brain-dead cadaveric multiorgan donors within the Nordic network for Clinical Islet Transplantation Laboratory in Uppsala, Sweden. Isolation and culturing conditions have been described previously [3], [4]. Briefly, the organs were dissociated mechanically and enzymatically using collagenase (Liberase, Roche, Indianapolis, IN). The dissociated tissue was then transferred to culture bags with CMRL 1066 medium (ICN Biomedicals, Costa Mesa, CA) supplemented with 10 mM Hepes (GIBCO BRL, Paisly, Scotland), 2 mM L-glutamin, 50 µg/ml Gentamycin, 0.25 µg/ml Fungizone (GIBCO BRL), 20 µg/ml Ciproxfloxacin (Bayer healthcare AG, Leverkusen, Germany) and 10 mM nicotinamide (Sigma-Aldrich, St. Louis, MO). The tissue was maintained at 37°C. Islets from four different donors were included and the least pure preparation had a purity of 96%. For three of the islet donors, exocrine tissue with a purity of more than 99% was also included. Tissues used for protein profiling on 44 different normal human tissue types were acquired from archives at the Department of Pathology of Uppsala University Hospital. TMAs were generated in accordance with strategies used in the Human Protein Atlas [5] and as previously described [6]. In brief, hematoxylin-eosin (HE) stained tissue sections from each formalin-fixed paraffin-embedded donor block were examined in order to determine the histology and select representative areas to sample for production of TMAs. Normal tissue was defined as microscopically normal and was most often selected from specimens collected from the vicinity of surgically removed tumors. An extended analysis of novel islet-specific proteins was performed on normal pancreatic tissue from 26 additional individuals, as well as seven individuals with T1D and seven individuals with T2D, all acquired within the Nordic Network for Clinical Islet Transplantation Laboratory in Uppsala, Sweden.

### Transcript profiling (RNA-seq)

Tissues samples used for RNA extraction were embedded in Optimal Cutting Temperature (O.C.T.) compound and stored at −80°C. An HE stained frozen section (4 µm) was prepared from each sample using a cryostat and the CryoJane Tape-Transfer System (Instrumedics, St. Louis, MO, USA, and examined by a pathologist (FP) to ensure proper tissue morphology. Three sections (10 µm) were cut from each frozen tissue block and the tissue was homogenized mechanically using a 3 mm steel grinding ball (VWR). Extraction of total RNA was performed using the RNeasy Mini Kit (Qiagen, Hilden, Germany) according to the manufacturer's instructions. Extracted RNA samples were analyzed using either an Experion automated electrophoresis system (Bio-Rad Laboratories, Hercules, CA, USA) with the standard-sensitivity RNA chip or an Agilent 2100 Bioanalyzer system (Agilent Biotechnologies, Palo Alto, USA) with the RNA 6000 Nano Labchip Kit. Only samples of high-quality RNA (RNA Integrity Number ≥7.5) were used for mRNA sequencing, performed on Illumina HiSeq2000/2500 machines (Illumina, San Diego, CA, USA) using the standard Illumina RNA-seq protocol with a read length of 2×100 bases. The samples were sequenced multiplexed 15 per lane, and the depth of sequencing was on average 30 million mappable reads for the two pancreatic tissue samples, 43 million reads for the four islet preparations and 34 million reads for the three exocrine preparations.

### Data analysis

Raw reads obtained from the sequencing system were trimmed for low quality ends with the software sickle [7], using a phred quality threshold of 20. All reads shorter than 54 bp after the trimmings were discarded. The processed reads were mapped to the GRCh37 version of the human genome with Tophat v2.0.3 [8], and potential PCR duplicates were eliminated with the MarkDuplicates module of Picard 1.77 [9]. To obtain quantification scores for all human genes, FPKM (fragments per kilobase of exon model per million mapped reads) values were calculated using Cufflinks v2.0.2 [10], which corrects for transcript length and the total number of mapped reads from the library to compensate for different read depths for different samples. The gene models from Ensembl build 69 were used in Cufflinks. All data was analyzed using R Statistical Environment, and a network analysis was performed using Cytoscape 3.0 [11]. For analyses where a log2-scale of the data was used, pseudo-counts of +1 were added to the data set.

### Barcode “Leakage”

As has been previously observed [12] multiplexing of samples on a single lane on the Illumina platform may in some cases lead to misidentification of barcodes, leading to what looks like a cross-contamination between samples. In the present investigation we observe a small quantity of misidentified reads (∼0.1%) for samples sequenced multiplexed on the same lane in the same run. This makes genes with very high expression in a certain tissue appear to have low expression in the other samples run on the same lane, which introduces a minor bias in the data. However, as most analyses here focus on relative differences (fold-changes), a leakage of 0.1% hardly affects the analyses.

### Specificity classification

The average FPKM value of all individual samples for each tissue was used to estimate the level of gene expression. A cutoff value of 1 FPKM was used as the detection limit [13]. Each of the 20,050 genes were classified into one of six categories based on the FPKM levels: (1) “Not detected” - <1 FPKM in pancreas; (2) “Pancreas enriched” – 5-fold higher FPKM level in pancreas compared to all other 26 tissues; (3) “Group enriched” – 5-fold higher FPKM level in a group of 2-7 tissues including pancreas compared to all other tissues; (4) “Expressed in all” – detected in all 27 tissues; (5) “Pancreas enhanced” – 5-fold higher FPKM level in pancreas compared to the average FPKM value of all 27 tissues and (6) “Mixed” – genes expressed in 1–26 tissues and in none of the above categories. Genes categorized as pancreas enriched, group enriched or pancreas enhanced were together defined as elevated in pancreas, and a “Pancreas-specific score” was calculated for each gene dividing the pancreas FPKM by the maximum FPKM value in any of the other 26 tissues.

### Gene ontology analysis

A gene ontology [14] analysis was performed using the GOrilla tool [15] in order to determine overrepresented GO categories in the gene set of tissue enriched genes. For the cellular component analysis the GOSlim GOA associations were used to determine whether genes encoded extracellular, intracellular or membrane bound proteins. The number of genes for each term was counted, allowing a gene to be associated with more than one term. A list of all genes analyzed in this study was used as the background list in GOrilla.

### Antibody-based profiling

TMAs were generated as previously described [6], containing triplicate 1 mm cores of 44 different types of normal tissues, including three cores of pancreatic tissue. Sections (4 µm thick) of the TMA blocks were deparaffinized in xylene, hydrated in graded alcohols and blocked for endogenous peroxidase in 0.3% hydrogen peroxide diluted in 95% ethanol. For antigen retrieval, a Decloaking chamber (Biocare Medical, Walnut Creek, CA) was used, immersing the slides in Citrate buffer, pH6 (Lab Vision, Freemont, CA) for 4 minutes at 125°C and then allowed to cool to 90°C. Automated immunohistochemistry was performed as previously described [6]. Validation of primary antibodies was performed as previously described [16], [17]. Incubation with PBS instead of primary antibody served as negative control. Details on the antibodies used in the examples, as well as quality control and validation of the antibodies are displayed in S1 Table. Immunohistochemically stained and mounted slides were scanned using an Aperio ScanScope XT Slide Scanner (Aperio Technologies, Vista, CA, USA) for generation of high-resolution digital images, followed by manual scoring of intensity and fraction of positive cells in different tissues by certified pathologists.

### Data availability

FPKM values for all tissue samples (not including the isolated islet and exocrine samples) will be available for downloads without any restrictions (www.proteinatlas.org/about/download). The primary data (reads) are available through the Array Express Archive (www.ebi.ac.uk/arrayexpress/) under the accession number: E-MTAB-1733. Transcript profiling data (FPKM values) for each gene in each cell and tissue type is available at the Human Protein Atlas website (www.proteinatlas.org), together with all immunohistochemistry data from the antibody-based profiling.

### Statistical analysis

Statistical evaluation was performed using the International Business Machines Corp. Statistical Package for the Social Sciences (IBM SPSS, Chicago, IL, USA). Independent samples Kruskal-Wallis test was used to test differences between groups.

## Results

### The transcriptomics analysis

Comparative transcriptome analysis by deep sequencing (RNA-Seq) of 27 different human tissues was conducted using fresh frozen tissues from altogether 95 individuals including two samples of pancreatic tissue, with the addition of four samples of isolated pancreatic islets and three samples of exocrine tissue. To quantify the transcriptome in each sample, normalized mRNA levels were calculated as FPKM values. An FPKM of 1 was set as cut-off for detection in these analyses, which roughly represents one mRNA per cell [13]. Using this cut-off, 61% of all protein coding genes were found to be expressed in the pancreas. The distribution of FPKM values in pancreas ranged from 1 FPKM up to 59,629, which yields a dynamic range of 10^5^ between the highest and lowest expressed genes. Highest expression was observed for the exocrine enzyme Chymotrypsinogen B1 (CTRB1), closely followed by a number of other digestive enzymes.

**Figure 1:**
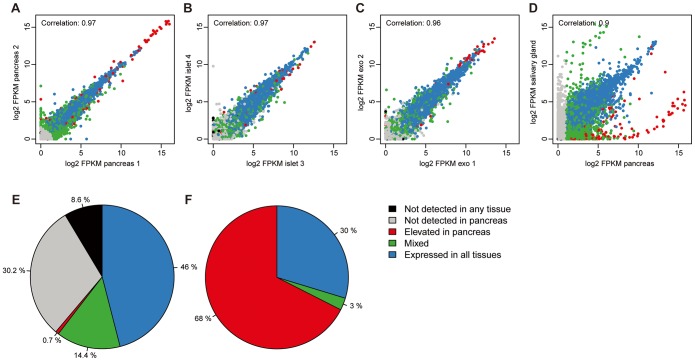
Sample correlations and classification of all human protein coding genes. Scatter plots of FPKM values for all detected genes in (A) two pancreas tissue samples, (B) two islet samples, (C) two exocrine samples (D) and pancreas and salivary gland. (E) Pie chart showing the classification of all genes in pancreas, based on transcript abundance and number of tissues with expression. (F) Pie chart showing the distribution of the expressed mRNA molecules in pancreas.

In order to determine the biological variance between pancreatic samples from different individuals, pairwise Spearman correlations were employed, plotting the expression levels of all protein coding genes. A high correlation (0.97) was observed between pancreas samples from the two different individuals (Fig. 1A). High correlations were also observed between the four isolated islet preparations (>0.96, Fig. 1B) and the three exocrine preparations (>0.94, Fig. 1C). The correlation between exocrine samples and whole pancreas was higher as compared with the correlation between islet preparations and whole pancreas (0.91 and 0.88 respectively, data not shown), not unexpected since the majority of cells in whole pancreas are exocrine glandular cells. The highest correlation with pancreas and other organs was salivary gland (0.90, Fig. 1D), while the lowest correlation was found between pancreas and testis (0.71, data not shown).

### The pancreas transcriptome

The transcriptomic analysis across 27 tissue types allowed for categorization of all the human protein-coding genes (n = 20,050) based on transcript abundance in pancreas (Fig. 1E). Altogether 61% of all genes were expressed in pancreas, with the largest class of genes (n = 5,626) representing “house-keeping” genes expressed in all tissues. The second class of genes (n = 1,761) showed a mixed pattern of expression, detected in 2–26 of the tissue types, while only 146 genes were defined as elevated in pancreas as compared with other organs. The 146 pancreas elevated genes (S2 Table) were further sub-classified into pancreas enriched genes (n = 47), group enriched genes (n = 45) and pancreas enhanced genes (n = 54). A GO-analysis of the genes elevated in pancreas indicated an over-representation of genes related to digestion (17 genes), regulation of peptide secretion (11 genes) and regulation of hormone secretion (11 genes). A majority (64%) of the gene products were located in the extracellular space, while 26% were part of the intracellular compartment and 10% were found in the membrane region.

An analysis of the expression levels of each gene expressed in the pancreas allowed for a calculation of the relative mRNA pool for each of the categories, as shown in Fig. 1F. Interestingly, the 146 genes elevated in pancreas, which comprised only 0.7% of the total number of the genes expressed in pancreas, corresponded to as much as 68% of the mRNA pool in pancreas. An analysis of the genes contributing to the pancreas transcriptome shows that the transcriptional activity in pancreas is mainly related to secretion of digestive enzymes.

### Analysis of genes enriched in islet and exocrine isolates

**Figure 2:**
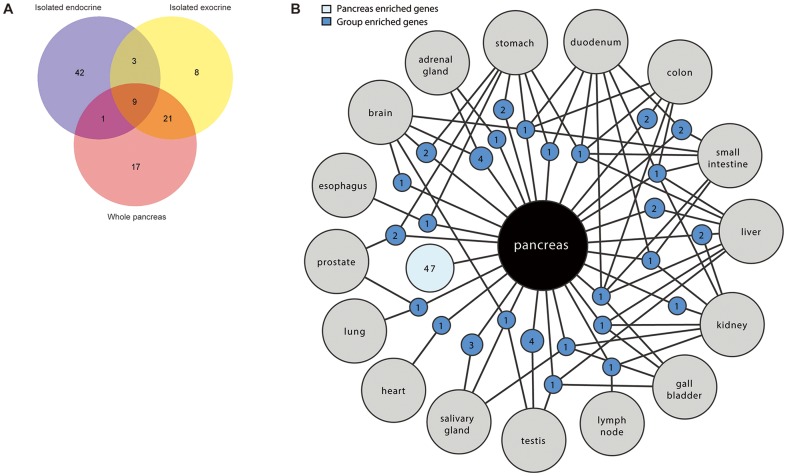
Genes enriched in pancreatic subcompartments and group enriched genes in pancreas. (A) Venn diagram visualizing the overlap of genes enriched in isolated islet samples (purple), isolated exocrine samples (yellow) and whole pancreas tissue (pink). (B) Network plot of the pancreas enriched and group enriched genes. Blue circle nodes represent a group of expressed genes and are connected to the respective enriched tissues (grey circles). The size of each blue node is related to the square root of the number of genes enriched in a particular combination of tissues.

The two main functions of the pancreas are carried out by specific cell types; islets of Langerhans and exocrine glandular cells. Anatomically, the islets of Langerhans are scattered within the organ, constituting only 1–2% of the total mass of pancreas and it can hence be anticipated that the expression of genes overrepresented in islet cells will be diluted in analyses of whole pancreas samples. This is not a problem when it comes to highly expressed islet hormones such as insulin and glucagon, however; low expressed islet genes could be undetected in the RNA-seq analysis using homogenized whole pancreas. With this background, a separate transcriptomic analysis was performed on isolated islet and exocrine preparations. The analysis resulted in 53 additional genes identified as enriched in the isolates that were not enriched in the whole pancreas samples (S3 Table). Of these 53 genes, 42 were enriched in the islet preparations and mainly associated with neuroendocrine function. Moreover, eight genes were enriched in exocrine preparations, associated with extracellular matrix and cell contact functions, while three genes were simultaneously enriched in both islet and exocrine preparations. In addition to the 53 genes enriched only in the islet and exocrine isolates, a number of genes were simultaneously enriched both in isolates and whole pancreas, while other genes were only enriched in whole pancreas and not in the isolates, as illustrated with a Venn diagram in Fig. 2A.

### Genes shared between pancreas and other tissues

A network plot was generated in order to illustrate genes simultaneously enriched in pancreas and other tissue types (Fig. 2B). Ten, eight and seven genes were shared with stomach, duodenum and small intestine respectively, dominated by genes with neuroendocrine functions or digestive functions. Eight genes were shared with kidney, mainly representing transport proteins associated with membrane permeability. Moreover, seven genes were shared with brain, reflecting a common neuroendocrine function, while five genes were shared with salivary gland, which is expected since the two organs have similar functions related to enzymatic secretion.

### Antibody-based profiling of genes elevated in pancreas

The genes elevated in pancreas identified by the transcriptomics analysis were further studied using antibody-based profiling utilizing immunohistochemistry data from the Human Protein Atlas project (www.proteinatlas.org) [5], for spatial analyses and determination of protein localization to different cell types within pancreas, including endocrine cells in islets of Langerhans, exocrine glandular cells and ductal cells. The protein profiling was based on immunohistochemically stained TMA cores from 44 different normal tissue types, including three cores of normal pancreas from different individuals.

### Proteins elevated in islets of Langerhans

The analysis of proteins selectively expressed in islets of Langerhans (Table 1) included several well-known hormones, such as insulin, glucagon, somatostatin and pancreatic polypeptide (PPY), identified as expressed in various endocrine cells (S1A Fig.). Other proteins selectively expressed in islets of Langerhans include transcription factors (NKX6-1 and PAX6) and proteins related to synapse formation (NPTX2), secretory processes (SCG5 and SCGN) and enzymatic activities (GAD2, PTPRN and IAPP (S1B Fig.).

**Table 1:** List of proteins elevated in islets of Langerhans, with information on mRNA expression, immunohistochemistry-based staining pattern and function.

Gene name	Description	Category RNA	Pancreas mean FPKM	Pancreas-specific score	Subcellular localization	Function
INS	Insulin	Pancreas enriched	2178.3	122.5	Cytoplasm	Lowering blood glucose
GCG	Glucagon	Pancreas enriched	701.4	6.6	Cytoplasm	Elevating blood glucose
SST	Somatostatin	Mixed (islet enriched)	170.0	N/A	Cytoplasm	Regulation of endocrine system
PPY	pancreatic polypeptide	Pancreas enriched	87.3	21.7	Cytoplasm	Regulation of pancreatic and gastrointestinal functions
NKX6-1	NK6 homeobox 1	Pancreas enhanced	2.9	0.6	Nucleus	Transcription regulation in beta cells
PAX6	paired box 6	Group enriched	7.7	0.3	Nucleus	Development and differentiation of alpha cells
NPTX2	neuronal pentraxin II	Mixed (islet enriched)	10.1	N/A	Cytoplasm	Excitatory synapse formation
SCG5	secretogranin V (7B2 protein)	Mixed (islet enriched)	64.7	N/A	Cytoplasm	Regulation of secretory pathways
SCGN	secretagogin, EF-hand calcium binding protein	Pancreas enhanced	30.8	1.4	Cytoplasm	Calcium influx and cell proliferation
GAD2	glutamate decarboxylase 2 (pancreatic islets and brain, 65kDa)	Brain enriched (islet enriched)	1.8	N/A	Cytoplasm	Autoantigen in diabetes
PTPRN	protein tyrosine phosphatase, receptor type, N	Group enriched	17.6	0.2	Cytoplasm	Autoantigen in diabetes
IAPP	islet amyloid polypeptide	Pancreas enriched	76.5	40.2	Cytoplasm	Inhibition of insulin-stimulated glucose utilization and glycogen deposition
CFC1	cripto, FRL-1, cryptic family 1	Group enriched	6.4	0.9	Cytoplasm	Embryonic development
FAM159B	family with sequence similarity 159, member B	Group enriched	5.5	0.8	Cytoplasm/membrane	Unknown
RBPJL	recombination signal binding protein for immunoglobulin kappa J region-like	Pancreas enriched	316.1	270.8	Cytoplasm	Putative transcription factor
RGS9	regulator of G-protein signaling 9	Mixed (islet enriched)	1.3	N/A	Cytoplasm	Regulation of dopamine/opioid signaling

In addition to proteins with well-known function in islets of Langerhans, four proteins selectively expressed in islet cells but previously not characterized in pancreas on the protein level were identified. The cryptic protein (CFC1) is suggested to be involved in embryonic development and neuronal patterning during gastrulation, however; the nearly exclusive expression in islet cells has not been described earlier. FAM159B, encoding a putative protein with unknown function and evidence of existence only at the transcript level, showed distinct expression in islet cells as well as in neuroendocrine cells of the stomach mucosa. The putative transcription factor recombining binding protein suppressor of hairless-like protein (RBPJL) not previously suggested to be expressed in pancreas showed a selective cytoplasmic expression pattern in islet cells. Another example is regulator of G-protein signaling 9 (RGS9), identified as enriched in only the islet isolates, was expressed in islet cells and also showed strong immunoreactivity in retinal photoreceptors (data not shown), well consistent with previous studies on phototransduction [18]. RGS9 has also been described in certain structures of the brain and is suggested to be involved in regulation of dopamine/opioid signaling, however, neither retina nor such brain structures were included in the present RNA-seq analysis.

### Protein expression of novel islet elevated proteins in diabetic and non-diabetic subjects

**Figure 3:**
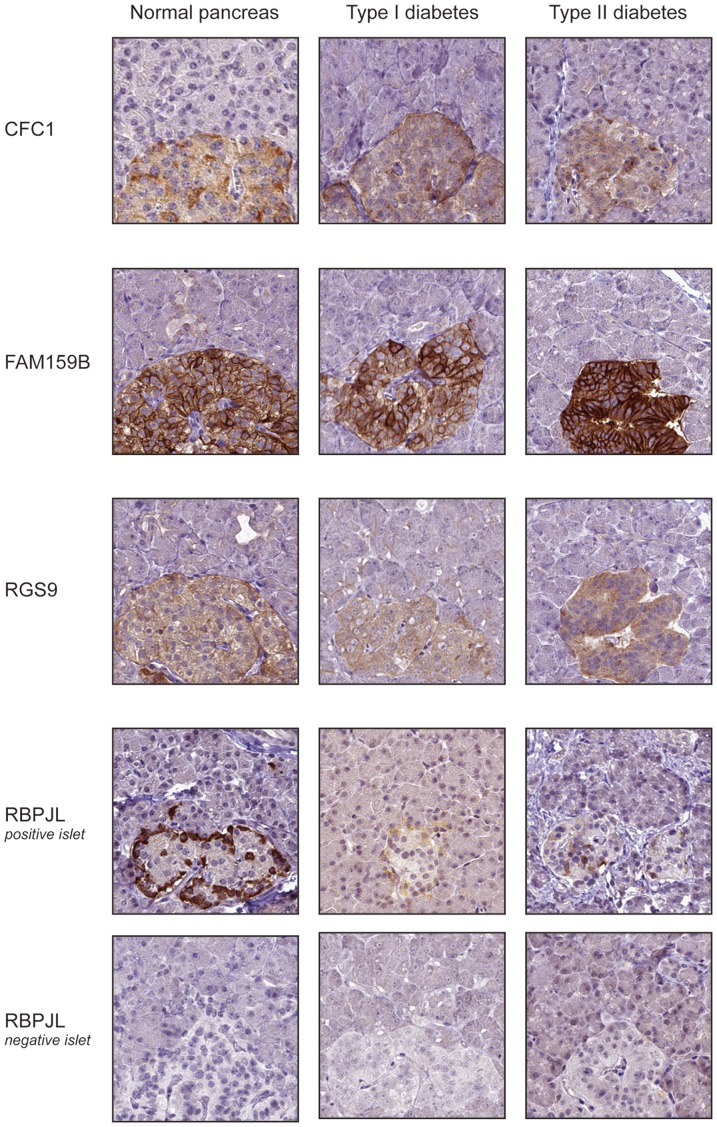
Immunohistochemical staining of proteins elevated in islets of Langerhans. Examples of four proteins (CFC1, FAM159B, RGS9 and RBPJL) not previously characterized in pancreas, with immunohistochemical staining pattern displayed in pancreas from normal subjects as well as patients with type I and type II diabetes. FAM159 showed cytoplasmic and membranous expression in the whole islet, while CFC1, RBPJL and RGS9 stained a subset of the islet cells. RBPJL was differentially expressed, with some patients being negative.

In order to investigate the specificity of the four novel proteins identified as selectively expressed in islets of Langerhans (CFC1, FAM159B, RBPJL and RGS9), further analysis was performed on a TMA containing pancreatic tissues from seven T1D subjects, seven T2D subjects as well as 26 non-diabetic subjects (Fig. 3). The expression of CFC1, FAM159B and RGS9 was consistent across the 40 analyzed samples, with RGS9 and CFC1 expressed in a subset of the islet cells in all diabetic and non-diabetic individuals, while FAM159B showed expression in a majority of the islet cells in all analyzed samples. Interestingly, RBPJL displayed a differential expression pattern, with distinct positivity in a small fraction of the islet cells in some individuals but not others. The expression was most frequent in non-diabetic subjects (63% positive) and T2D subjects (67% positive), while only 43% of the T1D subjects showed positivity, although the differences between groups did not prove to be significant (*p* = 0.531). The intensity of the RBPJL staining also varied between individuals, with strongest immunoreactivity observed in a few non-diabetic subjects.

### Proteins elevated in exocrine glandular cells

The exocrine part of pancreas comprises 98% of the total mass and is mainly involved in the production and secretion of digestive enzymes, utilizing a large proportion of the transcriptional activity in the pancreas. The main constituents of exocrine pancreas are glandular cells, i.e. acinar and ductal cells. Examples of proteins selectively expressed in exocrine glandular cells are listed in Table 2. Twelve of these examples include proteins known to be associated with enzymatic digestion (AMY2A, PNLIP, CEL, PLA2G1B, PRSS1, CELA3B, CPA1, CPB1, SPINK1, CTRL, GP2 and SYCN), displayed in S2A Fig.

**Table 2:** List of proteins elevated in exocrine glandular cells, with information on mRNA expression, immunohistochemistry-based staining pattern and function.

Gene name	Description	Category RNA	Pancreas mean FPKM	Pancreas-specific score	Subcellular localization	Function
AMY2A	amylase, alpha 2A (pancreatic)	Pancreas enriched	55123.6	533.9	Cytoplasm	Digestion of carbohydrates
PNLIP	pancreatic lipase	Pancreas enriched	31024.4	115.5	Cytoplasm	Hydrolyzation of lipids
CEL	carboxyl ester lipase (bile salt-stimulated lipase)	Pancreas enriched	16505.3	261.6	Cytoplasm	Catalyzation of lipids, absorption of vitamins
PLA2G1B	phospholipase A2, group IB (pancreas)	Pancreas enriched	14183.8	165.3	Cytoplasm	Hydrolyzation of phosphoglycerids
PRSS1	protease, serine, 1 (trypsin 1)	Pancreas enriched	52773.1	113.5	Cytoplasm	Trypsinogen, protease
CELA3B	chymotrypsin-like elastase family, member 3B	Pancreas enriched	19335.1	319.2	Cytoplasm	Elastase, protease
CPA1	carboxypeptidase A1 (pancreatic)	Pancreas enriched	23103.8	184.0	Cytoplasm	Zymogen inhibition
CPB1	carboxypeptidase B1 (tissue)	Pancreas enriched	19880.5	134.8	Cytoplasm	Serum marker for pancreatitis
SPINK1	serine peptidase inhibitor, Kazal type 1	Pancreas enriched	5105.9	7.6	Cytoplasm	Trypsin inhibitor
CTRL	chymotrypsin-like	Pancreas enriched	2452.4	273.7	Cytoplasm	Protease
GP2	glycoprotein 2 (zymogen granule membrane)	Pancreas enriched	8955.0	273.9	Cytoplasm	Zymogen granule, target of CD-specific pancreatic autoantibodies
SYCN	syncollin	Pancreas enriched	5166.5	411.5	Cytoplasm	Exocytosis, fusion of zymogen granules
BHLHA15	basic helix-loop-helix family, member a15	Group enriched	105.3	1.9	Nucleus	Transcriptional regulation of acinar cell function
REG1A	regenerating islet-derived 1 alpha	Group enriched	8784.7	2.6	Cytoplasm	Inhibition of calcium carbonate precipitation
PDIA2	protein disulfide isomerase family A, member 2	Pancreas enriched	957.0	15.5	Cytoplasm	Modulation estrogen levels in pancreas, folding of secretory proteins
AQP8	aquaporin 8	Group enriched	483.1	1.8	Membrane	Mediation of water transport across cell membranes
SLC38A5	solute carrier family 38, member 5	Pancreas enhanced	144.6	3.5	Membrane	Sodium-dependent amino acid transport
GNMT	glycine N-methyltransferase	Group enriched	101.8	0.8	Cytoplasm	Methylation of glycine
AQP12A	aquaporin 12A	Pancreas enriched	181.4	116.2	Cytoplasm	Mediation of water transport across cell membranes
DPEP1	dipeptidase 1 (renal)	Pancreas enhanced	242.6	0.8	Membrane	Kidney membrane enzyme, hydrolysation of dipeptides
GATM	glycine amidinotransferase (L-arginine:glycine amidinotransferase)	Group enriched	1127.9	1.2	Cytoplasm	Local creatine biosynthesis
ERP27	endoplasmic reticulum protein 27	Pancreas enriched	588.4	27.8	Cytoplasm	Protein disulfide isomerase
SFRP5	secreted frizzled-related protein 5	Pancreas enhanced	62.0	2.9	Cytoplasm	Regulation of cell growth and differentiation
CBS	cystathionine-beta-synthase	Pancreas enhanced	82.5	0.7	Cytoplasm	Enzyme protecting neurons against hypoxic injury

In addition to proteins involved in enzymatic function, several proteins with other functions that have previously been characterized in pancreas with selective expression in exocrine glandular cells were identified, as exemplified in S2B Fig. Examples include BHLHA15, REG1A, PDIA2, AQP8 and SLC38A5, all with well-known functions in the pancreas, and GNMT which is associated with methylation of glycine and previously shown to be expressed in the liver. Although the GNMT protein has also been identified in exocrine glandular cells in rat tissue [19], no previous studies have shown the cellular distribution in human pancreas.

**Figure 4:**
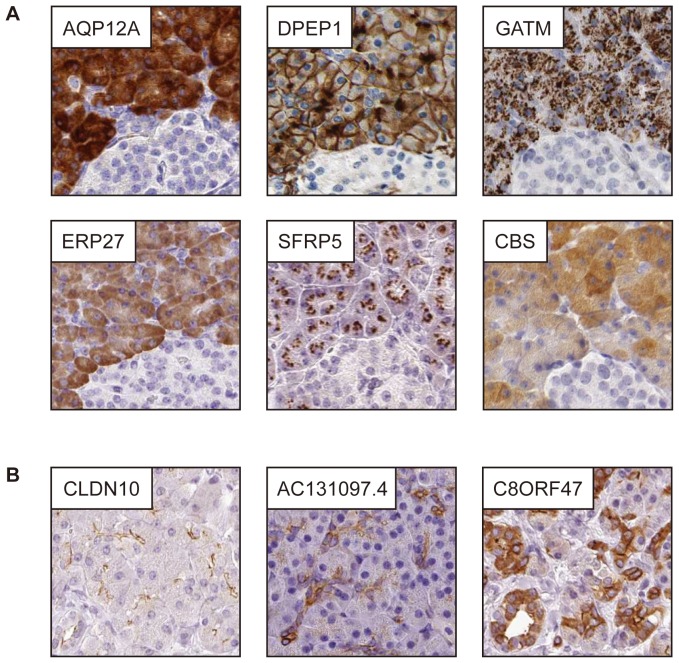
Immunohistochemical staining of proteins elevated in exocrine glandular cells and ductal cells. (A) Examples of six proteins not previously or only partly characterized in exocrine glandular cells. AQP12A, ERP27 and CBS revealed homogeneous cytoplasmic expression, while GATM and SFRP5 displayed a granular/dotlike pattern. DPEP1 showed distinct membranous positivity. (B) Examples of three proteins expressed in ductal cells, all showing distinct membranous positivity. C8ORF47 was expressed both in intercalated and interlobular ducts, while the positivity of CLDN10 and AC131097.4 was restricted to intercalated ducts.

Six proteins selectively expressed in pancreatic exocrine glandular cells, with no or only sparse previous information regarding the expression in pancreas, are displayed in Fig. 4A. Examples include: (i) The aquaporin family member AQP12A and although other aquaporins have been identified in human pancreas, the expression of AQP12A has only been described in mouse models. (ii) Dipeptidase 1 (DPEP1), a kidney membrane enzyme implicated in renal metabolism and hydrolysation of dipeptides, that showed a distinct membranous expression pattern in exocrine glandular cells, in addition to the brush border of renal tubules (iii) Glycine amidinotransferase (GATM), possibly involved in response to heart failure by elevating local creatine biosynthesis, with a distinct granular expression pattern in exocrine glandular cells, in addition to being highly expressed in kidney and liver. One earlier study on rats has shown that partial pancreatectomy resulted in downregulation of GATM [20]. (iv) ERP27, a member of the protein disulfide isomerase family of endoplasmic reticulum proteins and previously shown to be down-regulated in acute pancreatitis in rats [21], showed wide-spread cytoplasmic expression in acinar cells of human pancreas. (v) Secreted frizzled-related protein 5 (SFRP5) shown to regulate cell growth and differentiation, and to determine the polarity of photoreceptors in retina. Although pancreas has been suggested as a site for expression, the exact function in pancreas is unknown and the distinct granular expression pattern in exocrine glandular cells has previously not been described. (vi) Cystathionine-beta-synthase (CBS), an enzyme protecting neurons against hypoxic injury by regulating hydrogen sulfide, has previously been reported to be highly expressed in pancreas at the mRNA level [22]. The function of CBS is pancreas is unknown and the cellular distribution of CBS in the cytoplasm of exocrine glandular cells has not earlier been shown.

### Proteins elevated in ductal cells

The pancreatic ducts form a network of fluid-filled tubules connecting each acinar cell to duodenum. The main function of pancreatic ducts is to transport the digestive enzymes produced by exocrine glandular cells, and secrete HCO_3_^-^ rich fluid for regulation of the pH. Examples of proteins selectively expressed in ductal cells of pancreas are listed in Table 3. Well-known examples implicated in regulation of HCO_3_^-^ secretion include the cystic fibrosis transmembrane conductance regulator (CFTR) and the electrogenic sodium bicarbonate cotransporter 1 (SLC4A4) (S3 Fig.). Three less well-characterized proteins are CLDN10, AC131097.4 and C8ORF47 (Fig. 4B). The claudin 10 (CLDN10) protein is associated with tight junctions and calcium-dependent cell-adhesion activity. Although other claudins have been shown to be expressed in pancreas [23] and previous mRNA data suggests expression in pancreas (www.biogps.org), no literature describes the specific expression of CLDN10 in apical surfaces of intercalated ducts. Two genes, AC131097.4 and C8ORF47, both with evidence of existence only at the transcript level, encode for putative proteins that were selectively expressed in the pancreatic ductal system. The expression of AC131097.4 was restricted to intercalated ducts, while C8ORF47 was expressed both in intercalated and interlobular ducts.

**Table 3:** List of proteins elevated in ductal cells, with information on mRNA expression, immunohistochemistry-based staining pattern and function.

Gene name	Description	Category RNA	Pancreas mean FPKM	Pancreas-specific score	Subcellular localization	Function
CFTR	cystic fibrosis transmembrane conductance regulator (ATP-binding cassette sub-family C, member 7)	Pancreas enhanced	91.8	1.7	Membrane	Transport of Cl^-^, regulation of HCO3^-^ secretion
SLC4A4	solute carrier family 4, sodium bicarbonate cotransporter, member 4	Pancreas enhanced	136.5	1.3	Membrane	Regulation of HCO3^-^ and intracellular pH
CLDN10	claudin 10	Pancreas enhanced	138.0	0.9	Membrane	Tight junctions, calcium-dependent cell-adhesion activity
AC131097.4	Uncharacterized protein	Pancreas enhanced	9.0	1.9	Membrane	Unknown
C8ORF47	chromosome 8 open reading frame 47	Pancreas enhanced	11.5	1.2	Membrane	Unknown

## Discussion

Here, we describe the pancreas-specific proteome based on an integrative omics approach involving RNA-Seq data combined with detailed antibody-based profiling using immunohistochemistry, in order to determine the cell-specific protein localization in human pancreas. In addition to the two surgical pancreatic samples, isolated islets and exocrine tissue from organ donors were included in the analysis to enhance the probability to detect genes specifically expressed in islets of Langerhans. To our knowledge, this is the first study combining the transcriptomic analysis with protein profiling using intact normal human tissue to map expression of proteins relevant for pancreatic biology at a cellular resolution. In particular, we focused on proteins specifically expressed in islets of Langerhans, exocrine glandular cells and ductal cells.

The analysis of proteins elevated in pancreas identified a large number of well-known genes implicated in enzymatic and hormonal activities, but also several proteins hitherto not characterized in the context of pancreas. Some of these examples include CFC1, FAM159B, RBPJL and RGS9 expressed in islet cells, with FAM159B distinctly stained in the whole islet and CFC1, RBPJL and RGS9 expressed in a subset of the endocrine cells. The selective expression of these four proteins in islet cells as compared with exocrine glandular cells was confirmed on a larger set of both diabetic and non-diabetic individuals. Interestingly, RBPJL displayed a differential expression pattern in T1D, T2D and non-diabetic subjects, with some individuals being completely negative for RBPJL, suggesting that the expression is not restricted solely to a specific islet cell-type, but may rather reflect other characteristics or functionality that shared by different types of islet cells.

Beta cell transcriptomes have previously been analyzed to identify candidate genes involved in diabetes [24], [25], confirming that beta cell specific genes have neuronal-like properties. Moreover, 20% of the transcripts in beta cells were shown to be modified following exposure of inflammatory cytokines. These transcripts were mainly related to apoptosis and inflammatory processes, supporting the concept that the immune system is involved in T1D. Genome-wide association studies have also linked several loci with an increased risk of both T1D and T2D [26], [27]. In addition to studies focusing on beta cells, transcriptomic analyses have been performed on other major pancreatic cell types, such as alpha cells, acinar cells and ductal cells [28].

Diabetes affects almost 6% of the population and the incidence has shown to increase. If the trend continues, the number is believed to have doubled within the next 10-20 years [29]. Insulin injections establish glycemic control in diabetes patients, but microvascular and macrovascular complications are still an issue, resulting in lower life expectancy. One alternative therapy to obtain a more physiologic form of glycemic control is islet transplantation; however, a considerable proportion of the transplanted beta cells undergo apoptosis during the peritransplant period. A methodology studying the transplanted islets *in vivo*, such as imaging techniques, would aid in optimization of islet transplantation as well as other types of beta cell replacement therapies. Imaging of beta cells may also be useful in understanding the events that occur before onset of diabetes and allow for intervention strategies. Although a few candidate targets enabling imaging if the islets of Langerhans have been identified, few lack the specificity required for *in situ* imaging of beta cells [30]. Most importantly, a beta cell target should not be significantly expressed in other abdominal tissues or the exocrine pancreas [31], [32]. The present investigation focused on both whole pancreas tissue samples and isolated islets, comparing the expression in pancreas with mRNA and protein levels in a large number of other normal tissues. Hence, the list with genes enriched in islet preparations offers a unique possibility to identify novel molecules involved in endocrine functions. The four novel proteins identified in the present investigation as selectively expressed in islet cells (CFC1, FAM159B, RBPJL and RGS9) are unlikely to be specific for beta cells, as expression was observed in islets of both non-diabetic and T2D subjects as well as in islets of T1D subjects that lack beta cells. FAM159 may however serve as a potential marker for total islet mass.

An interesting observation is that a large majority (68%) of the transcripts in pancreas is encoded by elevated genes constituting only 0.7% of all genes expressed in pancreas, in contrast to almost all other tissue types for which genes with “housekeeping” functions dominate the mRNA pool [2]. This is well in line with the function of pancreas as a highly efficient secretory machinery, where the genes with highest expression levels encode for secreted proteins that excerpt their function in the extra-cellular space. Also in the exocrine compartments of pancreas, several proteins with unknown expression pattern and function in pancreas were identified, including GATM and SFRP5 displaying granular expression in exocrine glandular cells, and proteins encoded by the AC131097.4 and C8ORF47 genes, highly expressed in ductal cells.

Another disease associated with pancreas is pancreatitis, in which pancreatic enzymes are activated in pancreas instead of in the intestine, resulting in acute or chronic inflammation of the pancreas. Approximately 80% of cases with pancreatitis are due to alcohol abuse or cholelithiasis (gallstones), whereas the remaining 20% are associated with e.g. side effects from medications, mutations in genes encoding digestive enzymes [33], [34], trauma or autoimmune disorders. A rare form of chronic pancreatitis is autoimmune pancreatitis (AIP), a disease form that is essentially uncharacterized and the diagnosis and differentiation of AIP from pancreatic cancer is difficult [35]. Genes with elevated expression in pancreas and corresponding expression in the exocrine compartment of the pancreas could potentially be involved in various forms of pancreatitis and serve as starting points to explore possible biomarkers for differential diagnostics and treatment prediction.

A majority of pancreatic cancers are adenocarcinomas, originating from the ductal cells. As signs and symptoms appear in advanced stages of the disease, the overall prognosis for patients with pancreatic cancer is poor. Biomarkers for disease screening, risk stratification and prognosis, as well as prediction of therapy response and side effects would have a significant clinical impact, and a better understanding of the molecular constituents of normal pancreas can provide some of the basic knowledge needed to develop methods for improved diagnostics and treatment.

The analysis and repository of genes with elevated expression in the pancreas presented here provides a readily assessable and genome-wide knowledge base for further studies in pancreatic biology and disease. The results can be used for identifying potential biomarkers specifically expressed in various endocrine or exocrine compartments of pancreas. These biomarkers may be used as future targets for beta cell imaging, or identification, stratification or prognostication of patients with pancreatitis or pancreatic cancer. In summary, the present investigation presents a comprehensive resource of genes and proteins elevated in pancreas, where corresponding proteins have been further explored and localized to various subcompartments within pancreatic tissue at a single cell level.

## Supporting Information

S1 Fig **Immunohistochemical staining of proteins elevated in islets of Langerhans.** (A) Examples of four proteins (INS, GCG, SST and PPY) associated with hormonal function, showing cytoplasmic expression in different subsets of the islet cells. (B) Examples of eight proteins involved in transcriptional regulation, synapse formation, secretory processes and enzymatic activities. NKX6-1 and PAX6 displayed nuclear immunoreactivity, while the remaining proteins (NPTX2, SCG5, SCGN, GAD2, PTPRN and IAPP) revealed cytoplasmic positivity. IAPP was stained in a subset of the cells.
(PDF)

S2 Fig **Immunohistochemical staining of proteins elevated in exocrine glandular cells.** (A) Examples of 12 proteins associated with enzymatic digestion. CELA3B showed a secreted positivity, while the remaining eleven proteins (AMY2A, PNLIP, CEL, PLA2G1B, PRSS1, CPA1, CPB1, SPINK1, CTRL, GP2 and SYCN) were distinctly expressed in cytoplasm, with PRSS1 and GP2 displaying a slightly heterogenous pattern. (B) Examples of six proteins expressed in exocrine glandular cells with various well-known functions. BHLHA15 showed nuclear immunoreactivity, while AQP8 and SLC38A5 were distinctly expressed in membranes of acinar cells. REG1A, PDIA2 and GNMT displayed cytoplasmic positivity.
(PDF)

S3 Fig **Immunohistochemical staining of proteins elevated in ductal cells.** Examples of two proteins expressed in ductal cells (CFTR and SLC4A4), showing distinct membranous positivity in both intercalated and interlobular ducts.
(PDF)

S1 Table **Details on antibodies used in immunohistochemically stained examples in** **Figs. 3** **–** **4** **and Supplementary** **Figs. 1** **–** **3** **, including information on antibody validation.**
(DOCX)

S2 Table **List of 146 genes elevated in pancreas.**
(DOCX)

S3 Table **List of 53 genes enriched in isolated islets or exocrine preparations.**
(DOCX)
