# Supplementary figures and images for: The Human Pancreas Proteome Defined by Transcriptomics and Antibody-Based Profiling
Source: PLoS One. 2014 Dec 29;9(12):e115421. doi: 10.1371/journal.pone.0115421 (PMC4278897; doi:10.1371/journal.pone.0115421)

**A**

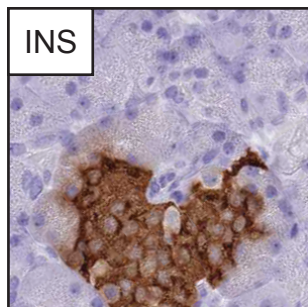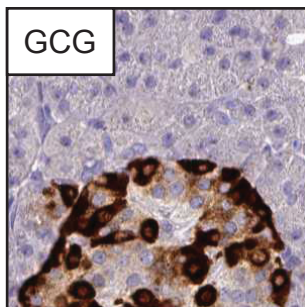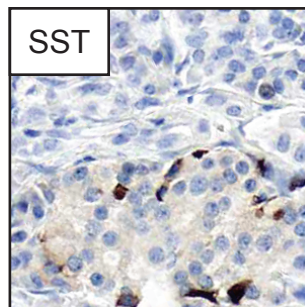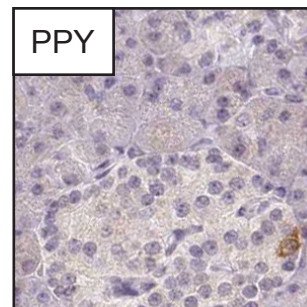

**B**

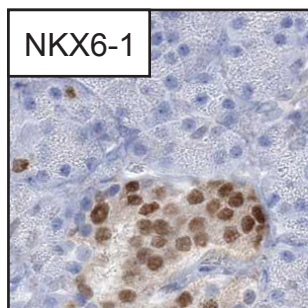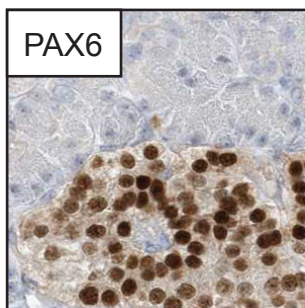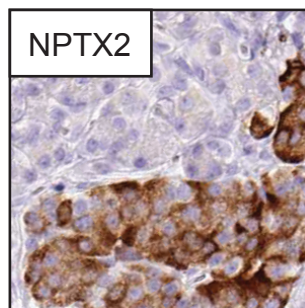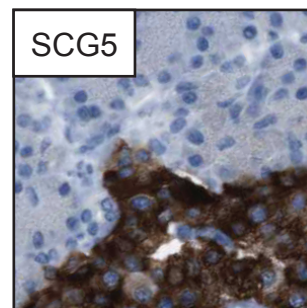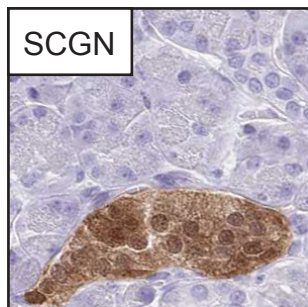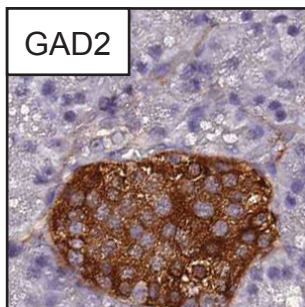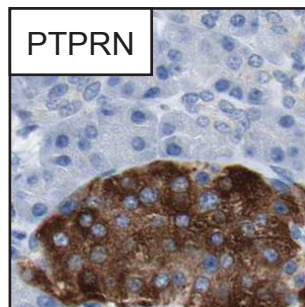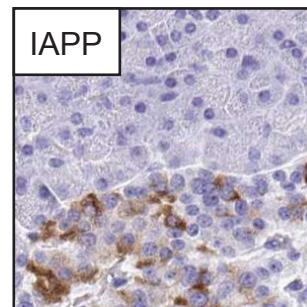

Supplement: S1 Fig — Immunohistochemical staining of proteins elevated in islets of Langerhans. (A) Examples of four proteins (INS, GCG, SST and PPY) associated with hormonal function, showing cytoplasmic expression in different subsets of the islet cells. (B) Examples of eight proteins involved in transcriptional regulation, synapse formation, secretory processes and enzymatic activities. NKX6-1 and PAX6 displayed nuclear immunoreactivity, while the remaining proteins (NPTX2, SCG5, SCGN, GAD2, PTPRN and IAPP) revealed cytoplasmic positivity. IAPP was stained in a subset of the cells. (PDF) [file pone.0115421.s001.pdf]

**A**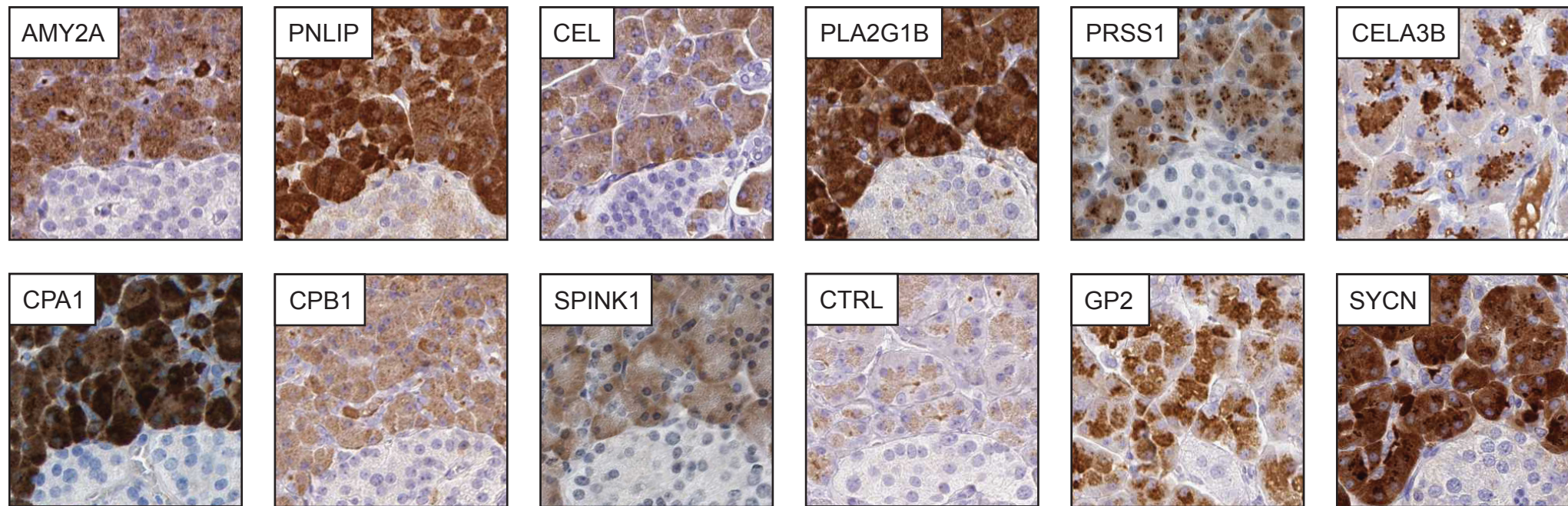**B**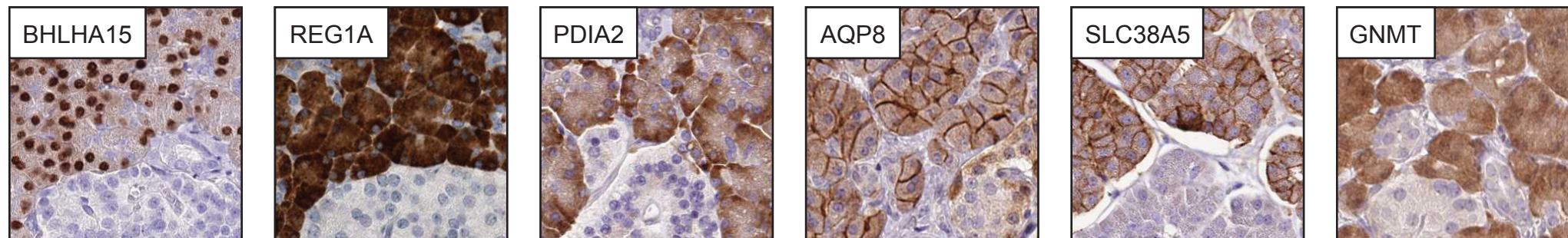

Supplement: S2 Fig — Immunohistochemical staining of proteins elevated in exocrine glandular cells. (A) Examples of 12 proteins associated with enzymatic digestion. CELA3B showed a secreted positivity, while the remaining eleven proteins (AMY2A, PNLIP, CEL, PLA2G1B, PRSS1, CPA1, CPB1, SPINK1, CTRL, GP2 and SYCN) were distinctly expressed in cytoplasm, with PRSS1 and GP2 displaying a slightly heterogenous pattern. (B) Examples of six proteins expressed in exocrine glandular cells with various well-known functions. BHLHA15 showed nuclear immunoreactivity, while AQP8 and SLC38A5 were distinctly expressed in membranes of acinar cells. REG1A, PDIA2 and GNMT displayed cytoplasmic positivity. (PDF) [file pone.0115421.s002.pdf]

CFTR

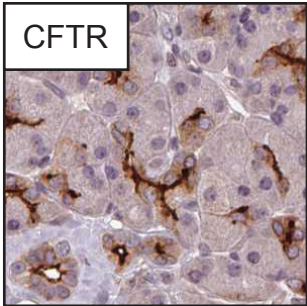

SLC4A4

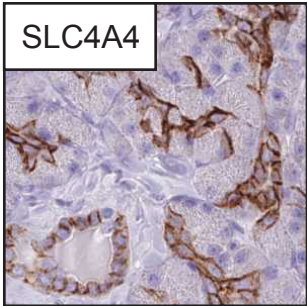

Supplement: S3 Fig — Immunohistochemical staining of proteins elevated in ductal cells. Examples of two proteins expressed in ductal cells (CFTR and SLC4A4), showing distinct membranous positivity in both intercalated and interlobular ducts. (PDF) [file pone.0115421.s003.pdf]
