# Supplementary material for: The Human Pancreas Proteome Defined by Transcriptomics and Antibody-Based Profiling
Source: PLoS One. 2014 Dec 29;9(12):e115421. doi: 10.1371/journal.pone.0115421 (PMC4278897; doi:10.1371/journal.pone.0115421)
Supplement: S1 Table — Details on antibodies used in immunohistochemically stained examples in Figs. 3 – 4 and Supplementary Figs. 1 – 3 , including information on antibody validation. (DOCX) [file pone.0115421.s004.docx]

**Supplementary Table 1.** Details on antibodies used in immunohistochemically stained examples in Fig 3-4 and Supplementary Figure 1-3, including information on antibody validation.

| **­­Gene name** | **Description** | **Antibody HPA ID^1^** | **Provider** | **Product name** | **Host species, clonality** | **Antibody dilution** | **Western blotting^2^** | **Specificity protein array^3^** | **Subcellular localization immune-flourescene^4^** | **Paired antibody similarity^5^** | **Immuno-histochemistry/RNA consistency^6^** |
| --- | --- | --- | --- | --- | --- | --- | --- | --- | --- | --- | --- |
| INS | insulin | HPA004932 | Atlas Antibodies AB | HPA004932 | Rabbit, pAb | 1/750 | Supportive | High | N/A | Similar | Consistent |
| GCG | glucagon | HPA036761 | Atlas Antibodies AB | HPA036761 | Rabbit, pAb | 1/800 | Not supportive | High | N/A | Similar | Consistent |
| SST | somatostatin | CAB000075 | DakoCytomation | A0566 | Rabbit, pAb | 1/4,000 | Not supportive | High | N/A | Similar | Consistent |
| PPY | pancreatic polypeptide | CAB016735 | BioPorto Diagnostics A/S | ABS 030-06 | Mouse, mAb | 1/60,000 | Uncertain | High | N/A | Similar | Consistent |
| NKX6-1 | NK6 homeobox 1 | HPA036774 | Atlas Antibodies AB | HPA036774 | Rabbit, pAb | 1/200 | Supportive | High | Supportive | N/A | Consistent |
| PAX6 | paired box 6 | HPA030775 | Atlas Antibodies AB | HPA030775 | Rabbit, pAb | 1/10 | Uncertain | High | Supportive | Similar | Consistent |
| NPTX2 | neuronal pentraxin II | HPA049799 | Atlas Antibodies AB | HPA049799 | Rabbit, pAb | 1/800 | Not supportive | Medium | N/A | Similar | Consistent |
| SCG5 | secretogranin V | HPA013136 | Atlas Antibodies AB | HPA013136 | Rabbit, pAb | 1/150 | Uncertain | High | N/A | N/A | Mainly consistent |
| SCGN | secretagogin, EF-hand calcium binding protein | CAB062563 | Atlas Antibodies AB | AMAB90630 | Mouse, mAb | 1/2,000 | Not supportive | High | N/A | Similar | Consistent |
| GAD2 | glutamate decarboxylase 2 | CAB002682 | Lab Vision/NeoMarkers | RB-9100 | Rabbit, pAb | 1/800 | Supportive | High | N/A | Partly similar | Mainly consistent |
| PTPRN | protein tyrosine phosphatase, receptor type, N | HPA007179 | Atlas Antibodies AB | HPA007179 | Rabbit, pAb | 1/250 | Uncertain | High | N/A | N/A | Consistent |
| IAPP | islet amyloid polypeptide | HPA053194 | Atlas Antibodies AB | HPA053194 | Rabbit, pAb | 1/1,200 | Uncertain | Medium | N/A | Similar | Consistent |
| CFC1 | cripto, FRL-1, cryptic family 1 | HPA041773 | Atlas Antibodies AB | HPA041773 | Rabbit, pAb | 1/1,500 | Uncertain | High | N/A | N/A | Mainly consistent |
| FAM159B | family with sequence similarity 159, member B | HPA011778 | Atlas Antibodies AB | HPA011778 | Rabbit, pAb | 1/25 | Uncertain | High | N/A | N/A | Mainly consistent |
| RBPJL | recombination signal binding protein for immunoglobulin kappa J region-like | CAB006846 | Chemicon | AB5790 | Rabbit, pAb | 1/2,000 | Not supportive | High | N/A | N/A | Consistent |
| RGS9 | regulator of G-protein signaling 9 | HPA014137 | Atlas Antibodies AB | HPA014137 | Rabbit, pAb | 1/250 | Not supportive | High | Uncertain | N/A | Mainly consistent |
| AMY2A | amylase, alpha 2A | CAB045960 | Origene | TA500146 | Mouse, mAb | 1/2,000 | Supportive | High | N/A | N/A | Consistent |
| PNLIP | pancreatic lipase | HPA062430 | Atlas Antibodies AB | HPA062430 | Rabbit, pAb | 1/1,300 | Uncertain | High | N/A | Similar | Consistent |
| CEL | carboxyl ester lipase | HPA052701 | Atlas Antibodies AB | HPA052701 | Rabbit, pAb | 1/500 | Uncertain | Medium | N/A | Similar | Consistent |
| PLA2G1B | phospholipase A2, group IB | HPA060803 | Atlas Antibodies AB | HPA060803 | Rabbit, pAb | 1/75 | Uncertain | High | N/A | Partly similar | Consistent |
| PRSS1 | protease, serine, 1 | CAB025538 | R&D Systems | MAB3848 | Mouse, mAb | 1/1,200 | Not supportive | High | Uncertain | Similar | Consistent |
| CELA3B | chymotrypsin-like elastase family, member 3A | HPA045650 | Atlas Antibodies AB | HPA045650 | Rabbit, pAb | 1/300 | Uncertain | High | N/A | N/A | Consistent |
| CPA1 | carboxypeptidase A1 | HPA021836 | Atlas Antibodies AB | HPA021836 | Rabbit, pAb | 1/1,500 | Supportive | High | N/A | Similar | Consistent |
| CPB1 | carboxypeptidase B1 | HPA046340 | Atlas Antibodies AB | HPA046340 | Rabbit, pAb | 1/4,500 | Supportive | Medium | N/A | Similar | Consistent |
| SPINK1 | serine peptidase inhibitor, Kazal type 1 | HPA027498 | Atlas Antibodies AB | HPA027498 | Rabbit, pAb | 1/750 | N/A | Medium | N/A | Partly similar | Mainly consistent |
| CTRL | chymotrypsin-like | HPA034504 | Atlas Antibodies AB | HPA034504 | Rabbit, pAb | 1/2,000 | Supportive | Medium | Uncertain | Similar | Consistent |
| GP2 | glycoprotein 2 | HPA016668 | Atlas Antibodies AB | HPA016668 | Rabbit, pAb | 1/1,500 | Supportive | High | N/A | Partly similar | Mainly consistent |
| SYCN | syncollin | HPA047654 | Atlas Antibodies AB | HPA047654 | Rabbit, pAb | 1/200 | Supportive | High | N/A | N/A | Consistent |
| BHLHA15 | basic helix-loop-helix family, member a15 | HPA047834 | Atlas Antibodies AB | HPA047834 | Rabbit, pAb | 1/200 | Supportive | High | N/A | Similar | Consistent |
| REG1A | regenerating islet-derived 1 alpha | CAB025138 | R&D Systems | MAB4937 | Rat, mAb | 1/500 | Not supportive | High | N/A | N/A | Consistent |
| PDIA2 | protein disulfide isomerase family A, member 2 | HPA053492 | Atlas Antibodies AB | HPA053492 | Rabbit, pAb | 1/1,000 | Not supportive | High | N/A | Similar | Consistent |
| AQP8 | aquaporin 8 | HPA046259 | Atlas Antibodies AB | HPA046259 | Rabbit, pAb | 1/700 | Not supportive | High | N/A | N/A | Mainly consistent |
| SLC38A5 | solute carrier family 38, member 5 | HPA047411 | Atlas Antibodies AB | HPA047411 | Rabbit, pAb | 1/200 | Uncertain | Medium | Uncertain | N/A | Mainly consistent |
| GNMT | glycine N-methyltransferase | HPA027501 | Atlas Antibodies AB | HPA027501 | Rabbit, pAb | 1/2,200 | Supportive | Medium | Supportive | N/A | Mainly consistent |
| AQP12A | aquaporin 12A | HPA042216 | Atlas Antibodies AB | HPA042216 | Rabbit, pAb | 1/75 | Not supportive | High | N/A | N/A | Consistent |
| DPEP1 | dipeptidase 1 | HPA009426 | Atlas Antibodies AB | HPA009426 | Rabbit, pAb | 1/2,000 | Supportive | High | N/A | Similar | Consistent |
| GATM | glycine amidinotransferase | HPA026077 | Atlas Antibodies AB | HPA026077 | Rabbit, pAb | 1/115 | Supportive | High | N/A | N/A | Mainly consistent |
| ERP27 | endoplasmic reticulum protein 27 | HPA039636 | Atlas Antibodies AB | HPA039636 | Rabbit, pAb | 1/3,000 | Supportive | High | N/A | Similar | Consistent |
| SFRP5 | secreted frizzled-related protein 5 | HPA019840 | Atlas Antibodies AB | HPA019840 | Rabbit, pAb | 1/600 | Supportive | High | Uncertain | N/A | Mainly not consistent |
| CBS | cystathionine-beta-synthase | HPA001223 | Atlas Antibodies AB | HPA001223 | Rabbit, pAb | 1/100 | Supportive | High | Supportive | N/A | Consistent |
| CFTR | cystic fibrosis transmembrane conductance regulator | HPA021939 | Atlas Antibodies AB | HPA021939 | Rabbit, pAb | 1/2,500 | Not supportive | High | N/A | Partly similar | Mainly consistent |
| SLC4A4 | solute carrier family 4 | HPA035628 | Atlas Antibodies AB | HPA035628 | Rabbit, pAb | 1/400 | Not supportive | High | N/A | Similar | Consistent |
| CLDN10 | claudin 10 | CAB012969 | Zymed | 38-8400 | Rabbit, pAb | 1/400 | Not supportive | High | N/A | Similar | Consistent |
| AC131097.4 | Protein LOC285095 | HPA043982 | Atlas Antibodies AB | HPA043982 | Rabbit, pAb | 1/225 | Not supportive | High | N/A | N/A | Mainly consistent |
| C8ORF47 | chromosome 8 open reading frame 47 | HPA025070 | Atlas Antibodies AB | HPA025070 | Rabbit, pAb | 1/3,200 | Uncertain | High | N/A | Partly similar | Consistent |

^1^www.proteinatlas.org

^2^ Western blotting: Supportive, uncertain or not supportive

^3^ Specificity on protein arrays: High, medium or low specificity

^4^ Subcellular localization based on immunofluorescence: Supportive or uncertain

^5^ Similarity to another antibody directed towards the same protein (paired antibody): Similar, partly similar or dissimilar

^6^ Similarity between immunohistochemical staining pattern and RNA-Seq data: Consistent, mainly consistent, mainly not consistent or not at all consistent
