# Supplementary material for: The Human Pancreas Proteome Defined by Transcriptomics and Antibody-Based Profiling
Source: PLoS One. 2014 Dec 29;9(12):e115421. doi: 10.1371/journal.pone.0115421 (PMC4278897; doi:10.1371/journal.pone.0115421)
Supplement: S2 Table — List of 146 genes elevated in pancreas. (DOCX) [file pone.0115421.s005.docx]

**Supplementary Table 2**. List of 146 genes elevated in pancreas.

| **Gene name** | **Description** | **Category RNA** | **Pancreas**  **mean FPKM** | **Pancreas- specific**  **score** |
| --- | --- | --- | --- | --- |
| CTRB2 | chymotrypsinogen B2 | Pancreas enriched | 51696.9 | 657.8 |
| PNLIPRP1 | pancreatic lipase-related protein 1 | Pancreas enriched | 4102.7 | 565.2 |
| CUZD1 | CUB and zona pellucida-like domains 1 | Pancreas enriched | 1008.2 | 537.7 |
| AMY2A | amylase, alpha 2A (pancreatic) | Pancreas enriched | 55123.6 | 533.9 |
| CELA2B | chymotrypsin-like elastase family, member 2B | Pancreas enriched | 8898.7 | 486.3 |
| CTRB1 | chymotrypsinogen B1 | Pancreas enriched | 59628.7 | 481.9 |
| SYCN | syncollin | Pancreas enriched | 5166.5 | 411.5 |
| CELA2A | chymotrypsin-like elastase family, member 2A | Pancreas enriched | 30856.7 | 328.5 |
| SERPINI2 | serpin peptidase inhibitor, clade I (pancpin), member 2 | Pancreas enriched | 622.9 | 327.5 |
| CELA3B | chymotrypsin-like elastase family, member 3B | Pancreas enriched | 19335.1 | 319.2 |
| CELA3A | chymotrypsin-like elastase family, member 3A | Pancreas enriched | 46684.3 | 313.9 |
| CTRC | chymotrypsin C (caldecrin) | Pancreas enriched | 14490.3 | 279.8 |
| GP2 | glycoprotein 2 (zymogen granule membrane) | Pancreas enriched | 8955.0 | 273.9 |
| CTRL | chymotrypsin-like | Pancreas enriched | 2452.4 | 273.7 |
| RBPJL | recombination signal binding protein for immunoglobulin kappa J region-like | Pancreas enriched | 316.1 | 270.8 |
| CEL | carboxyl ester lipase (bile salt-stimulated lipase) | Pancreas enriched | 16505.3 | 261.6 |
| CPA1 | carboxypeptidase A1 (pancreatic) | Pancreas enriched | 23103.8 | 184.0 |
| CPA2 | carboxypeptidase A2 (pancreatic) | Pancreas enriched | 9321.2 | 168.1 |
| AQP12B | aquaporin 12B | Pancreas enriched | 220.4 | 166.4 |
| PLA2G1B | phospholipase A2, group IB (pancreas) | Pancreas enriched | 14183.8 | 165.3 |
| CPB1 | carboxypeptidase B1 (tissue) | Pancreas enriched | 19880.5 | 134.8 |
| INS | insulin | Pancreas enriched | 2178.3 | 122.5 |
| AQP12A | aquaporin 12A | Pancreas enriched | 181.4 | 116.2 |
| PNLIP | pancreatic lipase | Pancreas enriched | 31024.4 | 115.5 |
| PRSS1 | protease, serine, 1 (trypsin 1) | Pancreas enriched | 52773.1 | 113.5 |
| CLPS | colipase, pancreatic | Pancreas enriched | 20285.4 | 97.8 |
| PRSS3 | protease, serine, 3 | Pancreas enriched | 8600.3 | 97.0 |
| IAPP | islet amyloid polypeptide | Pancreas enriched | 76.5 | 40.2 |
| CLPSL1 | colipase-like 1 | Pancreas enriched | 19.6 | 38.5 |
| G6PC2 | glucose-6-phosphatase, catalytic, 2 | Pancreas enriched | 26.8 | 28.6 |
| ERP27 | endoplasmic reticulum protein 27 | Pancreas enriched | 588.4 | 27.8 |
| KIRREL2 | kin of IRRE like 2 (Drosophila) | Pancreas enriched | 25.2 | 23.6 |
| PPY | pancreatic polypeptide | Pancreas enriched | 87.3 | 21.7 |
| AMY2B | amylase, alpha 2B (pancreatic) | Pancreas enriched | 10473.7 | 20.8 |
| PDIA2 | protein disulfide isomerase family A, member 2 | Pancreas enriched | 957.0 | 15.5 |
| PTF1A | pancreas specific transcription factor, 1a | Pancreas enriched | 14.2 | 14.3 |
| AP000892.1 | beta-secretase 1 isoform E | Pancreas enriched | 42.9 | 10.6 |
| GPHA2 | glycoprotein hormone alpha 2 | Pancreas enriched | 170.6 | 9.7 |
| PM20D1 | peptidase M20 domain containing 1 | Pancreas enriched | 43.1 | 8.2 |
| SPINK1 | serine peptidase inhibitor, Kazal type 1 | Pancreas enriched | 5105.9 | 7.6 |
| SLC30A8 | solute carrier family 30 (zinc transporter), member 8 | Pancreas enriched | 34.2 | 7.5 |
| GRPR | gastrin-releasing peptide receptor | Pancreas enriched | 9.1 | 7.2 |
| GCG | glucagon | Pancreas enriched | 701.4 | 6.6 |
| AC011298.2 | Uncharacterized protein | Pancreas enriched | 9.4 | 6.1 |
| COCH | coagulation factor C homolog, cochlin (Limulus polyphemus) | Pancreas enriched | 212.6 | 6.1 |
| FBXW12 | F-box and WD repeat domain containing 12 | Pancreas enriched | 3.2 | 5.7 |
| TMEM52 | transmembrane protein 52 | Pancreas enriched | 87.9 | 5.1 |
| TMED6 | transmembrane emp24 protein transport domain containing 6 | Group enriched | 131.6 | 4.6 |
| LHB | luteinizing hormone beta polypeptide | Pancreas enhanced | 5.7 | 3.9 |
| SLC38A5 | solute carrier family 38, member 5 | Pancreas enhanced | 144.6 | 3.5 |
| NRG4 | neuregulin 4 | Pancreas enhanced | 68.3 | 3.3 |
| CLPSL2 | colipase-like 2 | Pancreas enhanced | 2.6 | 3.0 |
| SFRP5 | secreted frizzled-related protein 5 | Pancreas enhanced | 62.0 | 2.9 |
| GPR150 | G protein-coupled receptor 150 | Pancreas enhanced | 2.0 | 2.8 |
| REG1A | regenerating islet-derived 1 alpha | Group enriched | 8784.7 | 2.6 |
| C8orf12 | chromosome 8 open reading frame 12 | Pancreas enhanced | 2.3 | 2.5 |
| SPAG4 | sperm associated antigen 4 | Pancreas enhanced | 50.1 | 2.5 |
| POMC | proopiomelanocortin | Pancreas enhanced | 10.0 | 2.4 |
| SYBU | syntabulin (syntaxin-interacting) | Pancreas enhanced | 100.3 | 2.4 |
| GPR119 | G protein-coupled receptor 119 | Pancreas enhanced | 1.1 | 2.3 |
| CNPY1 | canopy 1 homolog (zebrafish) | Pancreas enhanced | 1.2 | 2.3 |
| IL22RA1 | interleukin 22 receptor, alpha 1 | Pancreas enhanced | 55.2 | 2.2 |
| SCGB2B2 | secretoglobin, family 2B, member 2 | Pancreas enhanced | 3.1 | 2.2 |
| BCAT1 | branched chain amino-acid transaminase 1, cytosolic | Pancreas enhanced | 30.8 | 2.1 |
| ALDH1L2 | aldehyde dehydrogenase 1 family, member L2 | Pancreas enhanced | 21.7 | 2.0 |
| NUPR1 | nuclear protein, transcriptional regulator, 1 | Pancreas enhanced | 716.8 | 2.0 |
| FUT1 | fucosyltransferase 1 (galactoside 2-alpha-L-fucosyltransferase, H blood group) | Pancreas enhanced | 10.5 | 2.0 |
| BHLHA15 | basic helix-loop-helix family, member a15 | Group enriched | 105.3 | 1.9 |
| HOMER2 | homer homolog 2 (Drosophila) | Pancreas enhanced | 124.2 | 1.9 |
| AC131097.4 | Uncharacterized protein | Pancreas enhanced | 9.0 | 1.9 |
| CELA1 | chymotrypsin-like elastase family, member 1 | Group enriched | 3.2 | 1.9 |
| AQP8 | aquaporin 8 | Group enriched | 483.1 | 1.8 |
| GUCA1C | guanylate cyclase activator 1C | Group enriched | 19.0 | 1.8 |
| CFTR | cystic fibrosis transmembrane conductance regulator (ATP-binding cassette sub-family C, member 7) | Pancreas enhanced | 91.8 | 1.7 |
| CTD-2600O9.1 | Uncharacterized protein | Pancreas enhanced | 2.0 | 1.6 |
| ABCC8 | ATP-binding cassette, sub-family C (CFTR/MRP), member 8 | Pancreas enhanced | 16.6 | 1.5 |
| TEX11 | testis expressed 11 | Group enriched | 19.0 | 1.5 |
| GLP1R | glucagon-like peptide 1 receptor | Pancreas enhanced | 3.0 | 1.5 |
| FFAR1 | free fatty acid receptor 1 | Pancreas enhanced | 1.3 | 1.5 |
| CLDN9 | claudin 9 | Pancreas enhanced | 2.1 | 1.4 |
| C2orf27B | chromosome 2 open reading frame 27B | Pancreas enhanced | 1.1 | 1.4 |
| SCTR | secretin receptor | Pancreas enhanced | 30.1 | 1.4 |
| SLC30A2 | solute carrier family 30 (zinc transporter), member 2 | Pancreas enhanced | 40.1 | 1.4 |
| SCGN | secretagogin, EF-hand calcium binding protein | Pancreas enhanced | 30.8 | 1.4 |
| ARSE | arylsulfatase E (chondrodysplasia punctata 1) | Pancreas enhanced | 54.1 | 1.3 |
| CHAC1 | ChaC, cation transport regulator homolog 1 (E. coli) | Group enriched | 27.1 | 1.3 |
| BRSK2 | BR serine/threonine kinase 2 | Pancreas enhanced | 49.2 | 1.3 |
| SLC4A4 | solute carrier family 4, sodium bicarbonate cotransporter, member 4 | Pancreas enhanced | 136.5 | 1.3 |
| NR5A2 | nuclear receptor subfamily 5, group A, member 2 | Pancreas enhanced | 33.1 | 1.3 |
| C8orf47 | chromosome 8 open reading frame 47 | Pancreas enhanced | 11.5 | 1.2 |
| VTCN1 | V-set domain containing T cell activation inhibitor 1 | Pancreas enhanced | 15.0 | 1.2 |
| RP11-422N16.3 | Uncharacterized protein | Pancreas enhanced | 1.6 | 1.2 |
| GATM | glycine amidinotransferase (L-arginine:glycine amidinotransferase) | Group enriched | 1127.9 | 1.2 |
| KLK1 | kallikrein 1 | Group enriched | 3153.7 | 1.1 |
| MYCL1 | v-myc myelocytomatosis viral oncogene homolog 1, lung carcinoma derived (avian) | Pancreas enhanced | 32.9 | 1.1 |
| POU6F2 | POU class 6 homeobox 2 | Pancreas enhanced | 2.7 | 1.1 |
| ERRFI1 | ERBB receptor feedback inhibitor 1 | Pancreas enhanced | 311.6 | 1.1 |
| RP11-481A20.11 | Uncharacterized protein | Pancreas enhanced | 2.8 | 1.1 |
| CABP7 | calcium binding protein 7 | Pancreas enhanced | 1.6 | 0.9 |
| CFC1 | cripto, FRL-1, cryptic family 1 | Group enriched | 6.4 | 0.9 |
| C1orf127 | chromosome 1 open reading frame 127 | Pancreas enhanced | 2.5 | 0.9 |
| SCGB1C1 | secretoglobin, family 1C, member 1 | Pancreas enhanced | 1.2 | 0.9 |
| LHFPL5 | lipoma HMGIC fusion partner-like 5 | Group enriched | 1.3 | 0.9 |
| KCNK16 | potassium channel, subfamily K, member 16 | Group enriched | 7.7 | 0.9 |
| CLDN10 | claudin 10 | Pancreas enhanced | 138.0 | 0.9 |
| GNMT | glycine N-methyltransferase | Group enriched | 101.8 | 0.8 |
| MATN4 | matrilin 4 | Pancreas enhanced | 1.7 | 0.8 |
| FAM159B | family with sequence similarity 159, member B | Group enriched | 5.5 | 0.8 |
| DPEP1 | dipeptidase 1 (renal) | Pancreas enhanced | 242.6 | 0.8 |
| CCKBR | cholecystokinin B receptor | Group enriched | 10.5 | 0.8 |
| PAK3 | p21 protein (Cdc42/Rac)-activated kinase 3 | Pancreas enhanced | 11.0 | 0.8 |
| C2CD4B | C2 calcium-dependent domain containing 4B | Pancreas enhanced | 10.5 | 0.7 |
| CFC1B | cripto, FRL-1, cryptic family 1B | Group enriched | 3.2 | 0.7 |
| DNASE1 | deoxyribonuclease I | Group enriched | 116.0 | 0.7 |
| SPSB4 | splA/ryanodine receptor domain and SOCS box containing 4 | Pancreas enhanced | 6.9 | 0.7 |
| NEUROD1 | neuronal differentiation 1 | Group enriched | 4.5 | 0.7 |
| CAMK2N2 | calcium/calmodulin-dependent protein kinase II inhibitor 2 | Group enriched | 11.6 | 0.7 |
| CBS | cystathionine-beta-synthase | Pancreas enhanced | 82.5 | 0.7 |
| REG1B | regenerating islet-derived 1 beta | Group enriched | 325.5 | 0.7 |
| NPHS1 | nephrosis 1, congenital, Finnish type (nephrin) | Group enriched | 13.5 | 0.7 |
| NKX6-1 | NK6 homeobox 1 | Pancreas enhanced | 2.9 | 0.6 |
| AMY1C | amylase, alpha 1C (salivary) | Group enriched | 3224.4 | 0.6 |
| SLC39A5 | solute carrier family 39 (metal ion transporter), member 5 | Group enriched | 125.3 | 0.6 |
| ONECUT1 | one cut homeobox 1 | Group enriched | 6.7 | 0.6 |
| MYLK2 | myosin light chain kinase 2 | Pancreas enhanced | 3.1 | 0.6 |
| FOXA3 | forkhead box A3 | Group enriched | 20.7 | 0.5 |
| EGF | epidermal growth factor | Pancreas enhanced | 46.5 | 0.5 |
| LEFTY1 | left-right determination factor 1 | Group enriched | 41.9 | 0.5 |
| SARDH | sarcosine dehydrogenase | Group enriched | 18.6 | 0.4 |
| ADARB2 | adenosine deaminase, RNA-specific, B2 | Group enriched | 4.8 | 0.4 |
| ANKRD62 | ankyrin repeat domain 62 | Group enriched | 1.1 | 0.4 |
| TRPV6 | transient receptor potential cation channel, subfamily V, member 6 | Group enriched | 28.1 | 0.4 |
| PKHD1 | polycystic kidney and hepatic disease 1 (autosomal recessive) | Group enriched | 4.6 | 0.4 |
| FGL1 | fibrinogen-like 1 | Group enriched | 298.4 | 0.4 |
| NGB | neuroglobin | Group enriched | 2.4 | 0.4 |
| FXYD2 | FXYD domain containing ion transport regulator 2 | Group enriched | 282.0 | 0.3 |
| SLC17A4 | solute carrier family 17 (sodium phosphate), member 4 | Group enriched | 7.8 | 0.3 |
| REG3G | regenerating islet-derived 3 gamma | Group enriched | 23.7 | 0.3 |
| PAX6 | paired box 6 | Group enriched | 7.7 | 0.3 |
| GJD2 | gap junction protein, delta 2, 36kDa | Group enriched | 1.7 | 0.3 |
| RP11-664D7.4 | HCG1787533; Uncharacterized protein | Group enriched | 2.1 | 0.3 |
| C14orf105 | chromosome 14 open reading frame 105 | Group enriched | 19.3 | 0.2 |
| SPACA3 | sperm acrosome associated 3 | Group enriched | 10.8 | 0.2 |
| HNF1A | HNF1 homeobox A | Group enriched | 3.5 | 0.2 |
| INSM1 | insulinoma-associated 1 | Group enriched | 1.3 | 0.2 |
| PTPRN | protein tyrosine phosphatase, receptor type, N | Group enriched | 17.6 | 0.2 |
