# Supplementary material for: The Human Pancreas Proteome Defined by Transcriptomics and Antibody-Based Profiling
Source: PLoS One. 2014 Dec 29;9(12):e115421. doi: 10.1371/journal.pone.0115421 (PMC4278897; doi:10.1371/journal.pone.0115421)
Supplement: S3 Table — List of 53 genes enriched in isolated islets or exocrine preparations. (DOCX) [file pone.0115421.s006.docx]

**Supplementary Table 3**. List of 53 genes enriched in isolated islets or exocrine preparations.

| **Gene name** | **Description** | **Enriched compartment** | **Islet**  **mean**  **FPKM** | **Exocrine mean FPKM** | **Whole pancreas FPKM** |
| --- | --- | --- | --- | --- | --- |
| SST | somatostatin | Islet | 3573.6 | 343.2 | 170.0 |
| SCG5 | secretogranin V (7B2 protein) | Islet | 1367.4 | 175.3 | 64.7 |
| SCGN | secretagogin, EF-hand calcium binding protein | Islet | 455.9 | 40.0 | 30.9 |
| PTPRN | protein tyrosine phosphatase, receptor type, N | Islet | 445.3 | 43.5 | 17.6 |
| PCSK2 | proprotein convertase subtilisin/kexin type 2 | Islet | 336.5 | 35.5 | 10.8 |
| PCSK1 | proprotein convertase subtilisin/kexin type 1 | Islet | 265.8 | 23.5 | 4.6 |
| SVOP | SV2 related protein homolog (rat) | Islet | 244.5 | 8.8 | 1.3 |
| NPTX2 | neuronal pentraxin II | Islet | 206.1 | 25.4 | 10.1 |
| PAX6 | paired box 6 | Islet | 201.9 | 31.9 | 7.7 |
| CRYBA2 | crystallin, beta A2 | Islet | 182.4 | 9.9 | 5.5 |
| ADCYAP1 | adenylate cyclase activating polypeptide 1 (pituitary) | Islet | 170.2 | 14.5 | 3.2 |
| ABCC8 | ATP-binding cassette, sub-family C (CFTR/MRP), member 8 | Islet | 163.1 | 21.7 | 16.6 |
| MIR7-3HG | MIR7-3 host gene (non-protein coding) | Islet | 143.3 | 34.3 | 7.6 |
| GAD2 | glutamate decarboxylase 2 (pancreatic islets and brain, 65kDa) | Islet | 112.7 | 15.0 | 1.8 |
| CFC1 | cripto, FRL-1, cryptic family 1 | Islet | 79.3 | 5.5 | 6.4 |
| NEUROD1 | neuronal differentiation 1 | Islet | 70.3 | 6.4 | 4.5 |
| KCNK16 | potassium channel, subfamily K, member 16 | Islet | 59.6 | 2.3 | 7.8 |
| UCN3 | urocortin 3 | Islet | 57.9 | 11.0 | 3.6 |
| CFC1B | cripto, FRL-1, cryptic family 1B | Islet | 55.7 | 3.8 | 3.2 |
| HMGCLL1 | 3-hydroxymethyl-3-methylglutaryl-CoA lyase-like 1 | Islet | 54.6 | 5.7 | 0.9 |
| LOXL4 | lysyl oxidase-like 4 | Islet | 46.4 | 4.2 | 2.6 |
| GJD2 | gap junction protein, delta 2, 36kDa | Islet | 45.9 | 2.6 | 1.8 |
| RGS9 | regulator of G-protein signaling 9 | Islet | 45.2 | 5.0 | 1.3 |
| NKX6-1 | NK6 homeobox 1 | Islet | 43.9 | 3.4 | 2.9 |
| INSM1 | insulinoma-associated 1 | Islet | 36.6 | 2.5 | 1.3 |
| CABP7 | calcium binding protein 7 | Islet | 32.3 | 3.2 | 1.6 |
| LMO1 | LIM domain only 1 (rhombotin 1) | Islet | 24.5 | 1.8 | 0.7 |
| MYO3A | myosin IIIA | Islet | 24.1 | 3.0 | 0.3 |
| GCK | glucokinase (hexokinase 4) | Islet | 21.5 | 1.5 | 0.6 |
| C1orf127 | chromosome 1 open reading frame 127 | Islet | 21.4 | 1.2 | 2.5 |
| LRRC10B | leucine rich repeat containing 10B | Islet | 18.0 | 1.1 | 0.4 |
| MAFA | v-maf musculoaponeurotic fibrosarcoma oncogene homolog A (avian) | Islet | 17.3 | 0.5 | 0.8 |
| KCNJ6 | potassium inwardly-rectifying channel, subfamily J, member 6 | Islet | 16.0 | 1.2 | 0.3 |
| GLP1R | glucagon-like peptide 1 receptor | Islet | 16.0 | 1.0 | 3.0 |
| CNGA3 | cyclic nucleotide gated channel alpha 3 | Islet | 13.8 | 0.8 | 0.2 |
| GPR119 | G protein-coupled receptor 119 | Islet | 9.7 | 1.1 | 1.1 |
| GLRA1 | glycine receptor, alpha 1 | Islet | 8.8 | 0.2 | 0.3 |
| SIX3 | SIX homeobox 3 | Islet | 7.3 | 0.2 | 0.2 |
| SLC38A8 | solute carrier family 38, member 8 | Islet | 6.1 | 0.2 | 0.0 |
| KCNG3 | potassium voltage-gated channel, subfamily G, member 3 | Islet | 5.9 | 0.7 | 0.3 |
| GHSR | growth hormone secretagogue receptor | Islet | 4.2 | 0.3 | 0.0 |
| OLIG3 | oligodendrocyte transcription factor 3 | Islet | 0.6 | 0.0 | 0.1 |
| REG1B | regenerating islet-derived 1 beta | Exocrine | 1017.0 | 9106.6 | 325.5 |
| TXNRD1 | thioredoxin reductase 1 | Exocrine | 302.3 | 494.2 | 9.5 |
| SERPINA3 | serpin peptidase inhibitor, clade A (alpha-1 antiproteinase, antitrypsin), member 3 | Exocrine | 284.8 | 4028.8 | 245.8 |
| MMP1 | matrix metallopeptidase 1 (interstitial collagenase) | Exocrine | 281.6 | 524.2 | 0.2 |
|  |  |  |  |  |  |
| LAMC2 | laminin, gamma 2 | Exocrine | 48.5 | 316.4 | 1.6 |
| ITGA2 | integrin, alpha 2 (CD49B, alpha 2 subunit of VLA-2 receptor) | Exocrine | 19.9 | 91.4 | 0.8 |
| REG3G | regenerating islet-derived 3 gamma | Exocrine | 13.6 | 244.1 | 23.7 |
|  |  |  |  |  |  |
| KRTAP2-3 | keratin associated protein 2-3 | Exocrine | 0.2 | 0.9 | 0.0 |
| SRXN1 | sulfiredoxin 1 | Islet + exocrine | 263.5 | 311.3 | 6.1 |
| IL11 | interleukin 11 | Islet + exocrine | 129.1 | 140.9 | 0.2 |
| C2CD4A | C2 calcium-dependent domain containing 4A | Islet + exocrine | 64.2 | 10.6 | 1.3 |
